# Regional and local environmental conditions do not shape the response to warming of a marine habitat-forming species

**DOI:** 10.1038/s41598-017-05220-4

**Published:** 2017-07-11

**Authors:** C. Crisci, J.-B. Ledoux, K. Mokhtar- Jamaï, M. Bally, N. Bensoussan, D. Aurelle, E. Cebrian, R. Coma, J.- P. Féral, M. La Rivière, C. Linares, P. López-Sendino, C. Marschal, M. Ribes, N. Teixidó, F. Zuberer, J. Garrabou

**Affiliations:** 1 Polo de Desarrollo Universitario Modelización y Análisis de Recursos Naturales, Centro Universitario Regional del Este, Universidad de la República, Rocha, 27000 Uruguay; 2 CIIMAR/CIMAR, Centro Interdisciplinar de Investigação Marinha e Ambiental, Universidade do Porto, Porto, 4050-123 Portugal; 3 Institute of Marine Sciences (ICM-CSIC), Barcelona, 08003 Spain; 4 Aix Marseille Université, CNRS, IRD, Avignon Université, IMBE UMR 7263, Station Marine d’Endoume, Marseille, 13007 France; 5 Aix-Marseille Université, Mediterranean Institute of Oceanography (M I O), Université de Toulon, CNRS/IRD, Marseille, France; 6 IPSO FACTO, SCOPArl, Pole Océanologie, Marseille, 13001 France; 7 Centre for Advanced Studies of Blanes (CEAB-CSIC), Blanes, 17300 Spain; 8 Departament de Ciències Ambientals, Facultat de Ciències, Universitat de Girona, Girona, 17071 Spain; 9 Departament d’Ecologia, Universitat de Barcelona, Barcelona, 08028 Spain; 10 Stazione Zoologica Anton Dohrn, Villa Dohrn-Benthic Ecology Center, Punta San Pietro, Ischia, Naples, 80077 Italy; 11 Institut Pytheas, UMS 3470, CNRS Marseille, 13013 France

## Abstract

The differential response of marine populations to climate change remains poorly understood. Here, we combine common garden thermotolerance experiments in aquaria and population genetics to disentangle the factors driving the population response to thermal stress in a temperate habitat-forming species: the octocoral *Paramuricea clavata*. Using eight populations separated from tens of meters to hundreds of kilometers, which were differentially impacted by recent mortality events, we identify 25 °C as a critical thermal threshold. After one week of exposure at this temperature, seven of the eight populations were affected by tissue necrosis and after 30 days of exposure at this temperature, the mean % of affected colonies increased gradually from 3 to 97%. We then demonstrate the weak relation between the observed differential phenotypic responses and the local temperature regimes experienced by each population. A significant correlation was observed between these responses and the extent of genetic drift impacting each population. Local adaptation may thus be hindered by genetic drift, which seems to be the main driver of the differential response. Accordingly, conservation measures should promote connectivity and control density erosion in order to limit the impact of genetic drift on marine populations facing climate change.

## Introduction

The current global warming trend is affecting marine ecosystems in several ways, such as by decreasing ocean productivity, altering food web dynamics, shifting species distributions and reducing abundance^1–3^. Among these impacts, the increase in mass mortality events and disease outbreaks in marine species is particularly worrying because it severely threatens the structure and functioning of ecosystems and may significantly alter the provision of ecosystem services^4^. Generally, positive anomalies in seawater temperature have been concomitant with mass mortalities in affected regions^5–8^. These mortalities differentially affected species, populations and individuals at multiple spatial scales^7, 9–12^. For instance, field surveys conducted during mass mortality events in 1999 and 2003 in the Northwestern Mediterranean (NWM) revealed that, within the impacted species (mainly anthozoans and sponges), individuals separated by less than one meter showed varying responses ranging from severe to a complete absence of injuries while populations separated by a few to hundreds of kilometers displayed either similar or highly contrasting degrees of impact^7^. These differential responses might be explained by the complex interactions between populations and their local environments^11, 13–15^. In particular, environmental conditions, such as past and present thermal contexts, and biological processes, such as acclimatization or genetic adaptation, may be important drivers of the response of organisms to thermal stress^16, 17^. Accordingly, improving our understanding of the influences of environmental and biological factors on the response of populations to thermal stress is necessary to anticipate the consequences of climate change.

Acclimatization and local adaptation are two of the main biological processes modulating population responses to environmental variation, and they may drive the divergence in the phenotypic responses to thermal stress observed among populations^18–21^. Acclimatization implies physiological changes that allow an organism to maintain a functional phenotype when facing an environmental stressor^19, 21^, while local adaptation entails genetic changes driven by divergent selection^19, 20, 22^, which corresponds to spatial variations in natural selection resulting from local environmental conditions. Consequently, divergent selection may drive local populations to evolve traits that are advantageous in their local environments^18, 20^. Nevertheless, neutral evolutionary forces such as migration and genetic drift (i.e. the stochastic variations in alleles frequencies, caused by random sampling of genotypes and which is common in small and isolated populations)^23^ can also affect phenotypic divergence and thus influence differential responses. Migration can counteract divergent selection and limit local adaptation, while genetic drift may hinder local adaptation^18^.

The role of acclimatization and/or local adaptation to warming has been tested in various marine species through common garden thermotolerance experiments^24–26^ (see Materials and Methods section for a definition). For instance, differential acclimatization to thermal stress was demonstrated in crabs^27^, whereas local adaptation to thermal conditions has been suggested in corals^15^ and seagrasses^28^. Focusing on temperate octocorals, studies conducted on the Mediterranean red coral, *Corallium rubrum*, demonstrated the existence of differential adaptive abilities of populations depending on the depth of origin of the individuals and of their thermal history^29^. Local adaptation or acclimatization were considered to explain these results (see also refs 30, 31 and ref. 32 for other examples). However, these studies also suggested that genetic drift and acclimatization might play an important role in the phenotypic divergence between red coral populations by putatively modifying their abilities to adapt to local thermal environments.

**Figure 1:**
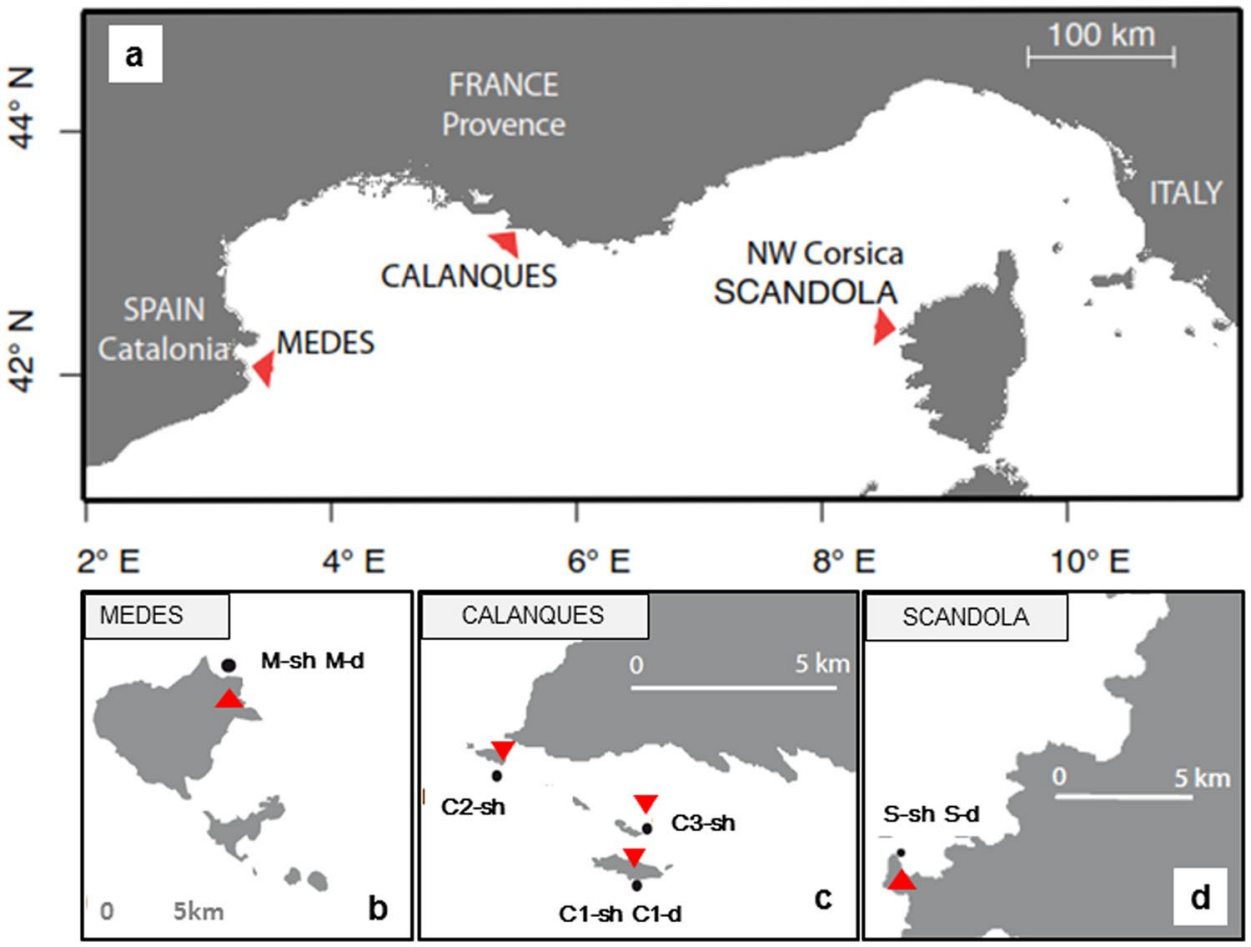
The study localities. The northwestern Mediterranean region with study localities (**a**). The study localities Medes (**b**), Calanques (**c**) and Scandola (**d**) and their populations: Medes shallow (M-sh, b) and Medes deep (M-d, b), Calanques 1, 2 and 3 shallow (C1-sh, C2-sh, C3-sh, c) and Calanques 1 deep (C1-d, c), Scandola shallow (S-sh, d) and Scandola deep (S-d, d). Figure 1a–d were performed with marmap R package^88^ (https://epante.wordpress.com/marmap/).

**Figure 2:**
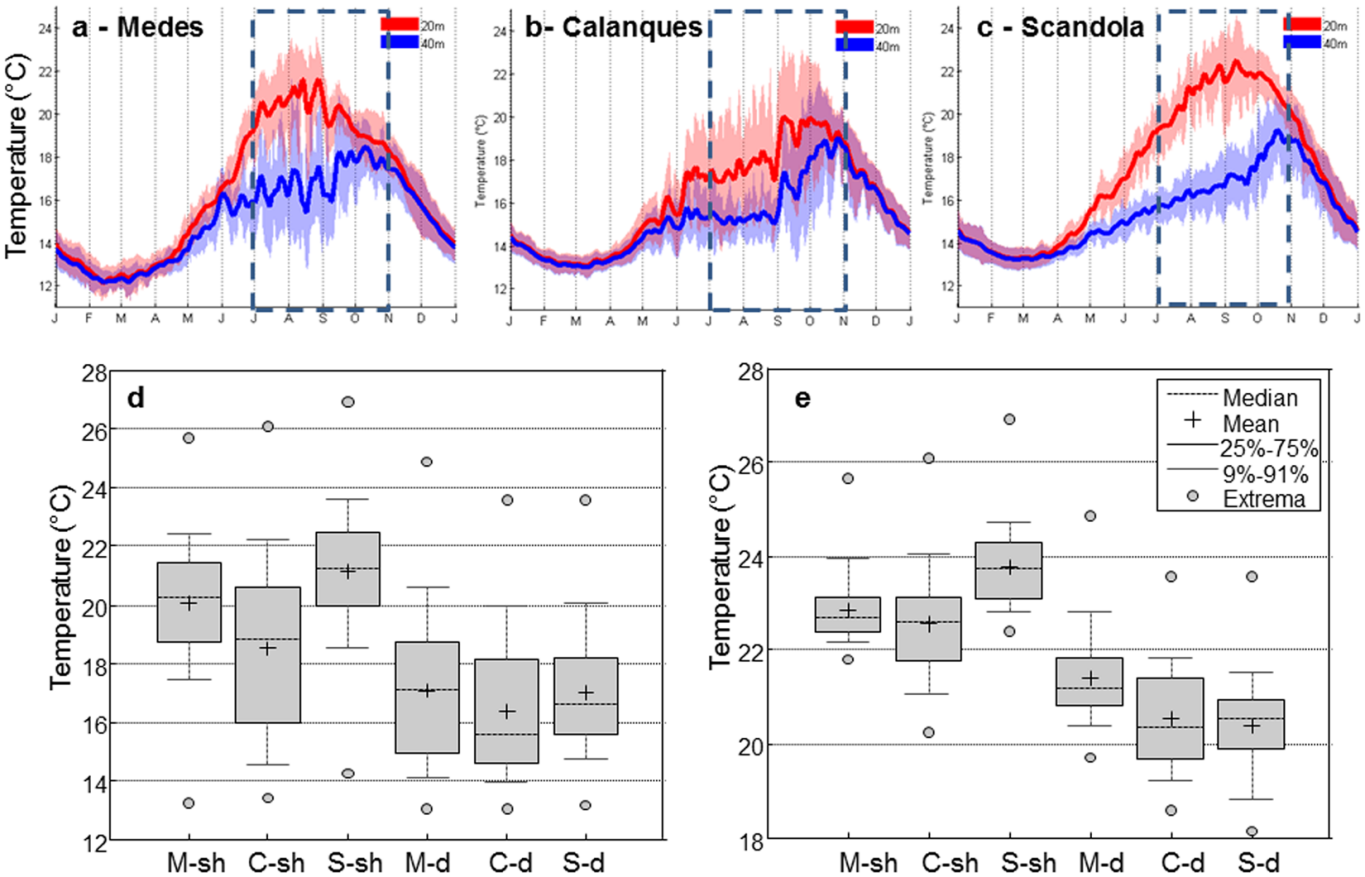
Temperature regimes of the study localities. Average annual temperature cycle (±SD) at 20 and 40 m depth for Medes (**a**), Calanques (Calanques 1) (**b**) and Scandola. Boxplots of hourly temperature records (of all available years) considering the July to October period and boxplots considering only the 10% of the data with the highest temperature are presented in (**d**) and (**e**) respectively. M-sh and M-d: Medes shallow and deep respectively, C-sh and C-d: Calanques 1 shallow and Calanques 1 deep respectively, S-sh and S-d: Scandola shallow and deep respectively.

In this study, we characterized the response to thermal stress of a temperate coral, the red gorgonian *Paramuricea clavata* (Risso, 1826) (Cnidaria, Anthozoa, Octocorallia). *P. clavata* is a key species of Mediterranean coralligenous assemblages and is one of the species most affected by recent mass mortality events^7, 33–35^. We carried out common garden experiments in aquaria using eight populations separated by tens of meters to hundreds of kilometers within the NWM (Fig. 1) and inhabiting contrasting temperature regimes at regional and local scales (ref. 36 and 37, subsection Temperature regimes of the study localities, Fig. 2). The aim of this study was twofold: 1) to acquire basic information about the thermotolerance features of *P. clavata* by monitoring colonies tissue necrosis at different temperatures (25, 26, 27 and 28 °C) in common garden experiments performed in aquaria; and 2) to evaluate the role of environmental conditions (particularly local thermal regimes) and biological processes (with a focus on local adaptation, genetic drift and acclimatization) in the differential responses of populations to thermal stress by combining colonies tissue necrosis data and population genetic analyses.

## Results

### Thermal regimes of the study localities and extreme hot conditions

The thermal regimes of the studied localities have been previously characterized^36, 37^. Important differences in the summer thermal regimes dynamics^36^ and in the characteristics of temperature anomalies associated with mass mortality events^37^ were revealed. Moreover, during these events, differences in temperature conditions were found as the main factor explaining the observed inter-regional variability in mortality impacts^37^.

In this study, local thermal regimes at depths at which common garden experiments colonies were sampled were documented through the analysis of 8 to 11 years of continuous (every hour) *in situ* temperature records.

Average annual temperature cycle (mean ± SD of daily T) at 20 and 40 m depths for Medes, Calanques and Scandola are shown in Fig. 2a–c respectively. The three sites share common features, typical for the NW-Mediterranean, starting by a strong seasonal component and significant vertical gradients from May-June to October-November (seasonal thermal stratification dynamics). Minima were recorded in February-March and maxima in August-September for 20 m depth and in October for 40 m depth. Overall, annual mean T ranged between 16.2–17.4 °C at 20 m and between 15.1–15.5 °C at 40 m depth. Considering the July to October period (hereafter referred to as warm period) notorious differences in thermal regimes (mean ± SD) between depths (inside localities) and between localities (same depth) (Fig. 2a–c) can be observed. Figure 2d provides additional information on the distribution of hourly temperature records for comparison across localities and depth levels considering the warm period.

First, it is worth noting the differences inside localities between the 20 and 40 m depth (warm period median temperature range 18.8–21.2 °C vs. 15.5–17.1 °C for 20 and 40 m depth respectively) (Fig. 2d). The most important vertical gradients were found at Scandola (∼4 °C), followed by Medes (∼3 °C) and then by Calanques (∼2 °C) (Fig. 2d).

At 20 m depth, Scandola is the warmest locality both considering the median (21.2 °C) and the maximum temperature (26.9 °C) (Fig. 2c,d). This locality also presented the most stable thermal regime with a narrow interquartile range of 2.3 °C (Fig. 2d). Coldest and most variable regimes were found in Calanques (Fig. 2b,d) presenting a median temperature of 18.8 °C, and interquartile range of 5 °C and clearly higher frequency of low temperature values (1^st^ quartile value of 16 °C) (Fig. 2d) which are a signature of the N winds driven by coastal upwelling of deep and cold waters at this locality. Intermediate conditions were found in Medes (Fig. 2a,d) where median and maximum temperatures were respectively 20.2 °C and 25.6 °C (Fig. 2a,d).

Even though at 40 m depth differences in mean temperature are attenuated, there are still clear patterns in thermal regimes. Medes is the warmest locality in depth, taking into account the median (17.1 °C) and maximum temperature values (24.9 °C) as well (Fig. 2d). Medes also experiences the most variable conditions during the warm period (Fig. 2a,d). For Calanques and Scandola, median values are respectively 15.5 °C and 16.7 °C; and maximum was 23.6 °C for both localities (Fig. 2d).

Finally, extreme hot conditions of the warm period were characterized. Figure 2e presents boxplots of the warm tail of hourly temperature records distribution (records over the 90^th^ percentile, considering the warm period of all available years). This representation of extreme temperatures reinforces the patterns observed on thermal regimes at seasonal scale in Fig. 2d. Differences from 1.4 to 3.4° Care observed between depths inside localities and of up to 1 °C between localities (considering mean of extreme temperatures) (Fig. 2e). At 20 m depth Scandola clearly experienced the most extreme temperatures, with no overlapping of Scandola interquartile range (23.1–24.3 °C) with respect to those of Medes and Calanques (Fig. 2e). In depth, Medes clearly present higher values of most extreme temperature values in comparison with Calanques and Scandola (Fig. 2e).

From a heat stress perspective, information analyzed here account for important differences in the thermal regimes and extreme hot temperatures distribution both among depths (inside localities) and between localities (same depths).

Indeed, global Kruskal-Wallis statistical test evidenced significant differences (p-value < 0.001) between temperature regimes (considering data of the warm period of all available years). Pair-wise comparisons (using Dunn-Sidak’s approach) accounted for significant differences among all pairs of localities and depths (all p-values < 0.01).

### Assessing the features of *Paramuricea clavata* thermotolerance

**Figure 3:**
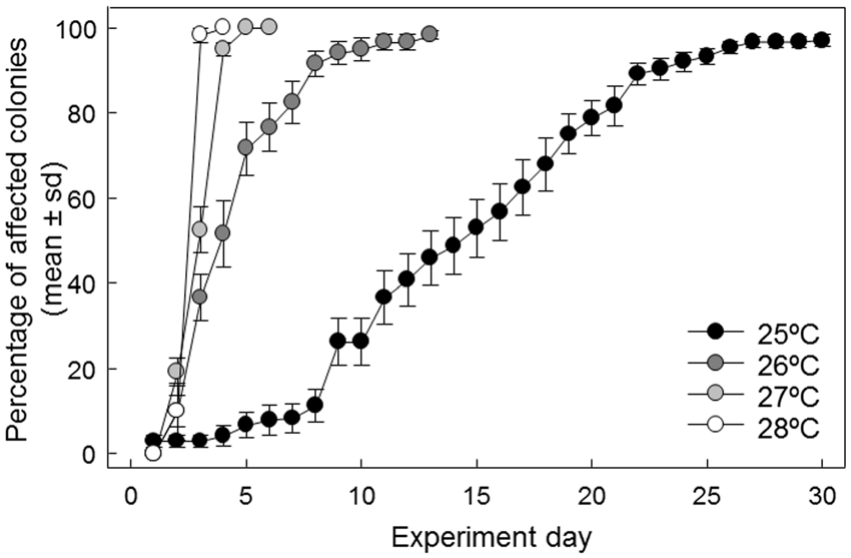
Results of the experiments to evaluate the thermotolerance thresholds of *P. clavata*. Daily mean (±SE) percentage of affected colonies (tissue necrosis >0%) for each temperature treatment: 25 °C, 26 °C, 27 °C and 28 °C. The 25 °C results correspond with the results of the ladder experiment from the day when T reached 25 °C. For clarification purposes, the mean (±SE) values of all tested populations (pooling sites and depths) are presented for each experiment.

We identified the critical temperature of *P. clavata* as 25 °C because after one week of exposure, 20 colonies from seven of the eight populations (8%) were affected by necrosis. After 30 days of exposure at 25 °C, the mean % of affected colonies increased gradually from 3% to 97% (Fig. 3).

Exposure to temperatures of 26, 27 and 28 °C resulted in a much more abrupt increase in necrosis, and a high level of mean affectation (90–100%) was observed after the 8^th^ day of the 26 °C experiment. For the 27 and 28 °C experiments, 90–100% mean affectation was reached on the 4^th^ and 3^rd^ days, respectively (Fig. 3). These results suggest that exposure to temperatures of 26 °C, or higher, for a short time is lethal to the red gorgonian *P. clavata*.

### Analyzing the potential factors that condition the response of populations to thermal stress

#### Importance of environmental factors

**Figure 4:**
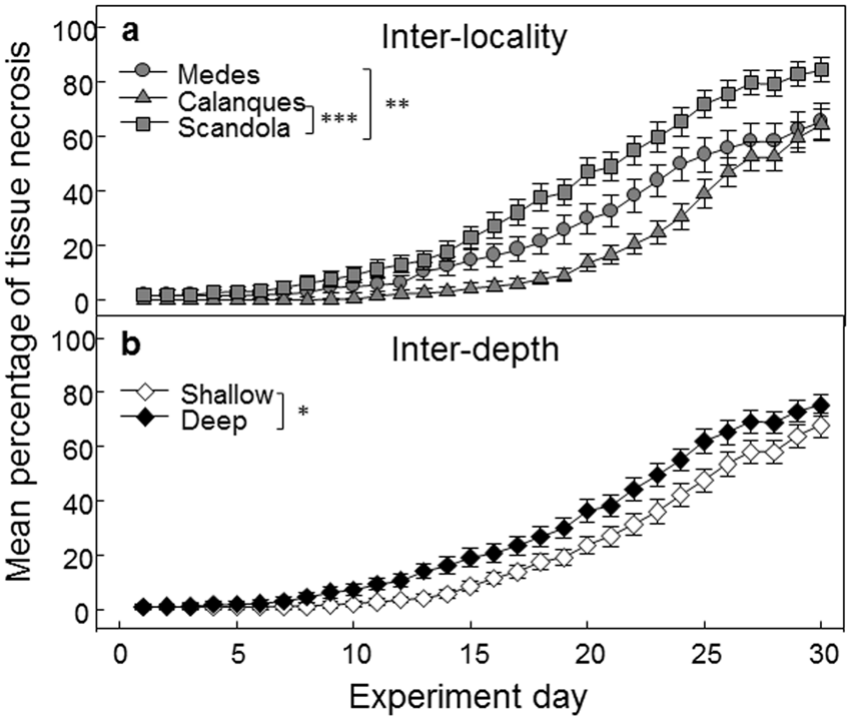
Results of the common garden experiment. Mean percentage (±SE) of tissue necrosis of colonies from the study localities (**a**) and depths (**b**) during the 25 °C experiment. The levels of significance of the differences between pairs of localities (**a**) and depths (**b**) from the two-way pairwise PERMANOVA are indicated as follows: ***p-value < 0.001, **p-value < 0.01, *p-value < 0.05. The lack of indications between pairs denotes the absence of significant differences.

Figure 4 illustrates the differences among localities (Fig. 4a) and depths (Fig. 4b) in the response to thermal stress (25 °C) of *P. clavata* populations dwelling in contrasting thermal regimes, and these results do not fully support our hypotheses. Considering the response to temperature of populations from different localities, the results were opposite to those expected. Scandola which was clearly the warmest locality at 20 m depth was the most affected while Calanques, which was the coldest locality both at 20 and 40 m depth (lowest median values), was the least affected (Figs 2 and 4a). Between depths, very little differences were observed being shallow populations less affected than the deep populations (Fig. 4b). The two-way multivariate PERMANOVA indicated significant results for the Locality (p-value < 0.001) and Depth factors (p-value < 0.05 but very close to the significance threshold: p-value = 0.049) while the interaction term (Locality x Depth) was not significant (p-value > 0.05) (Supplementary Table S1). Regarding localities, the pairwise post-hoc results of the two-way PERMANOVA indicated significant differences between the Medes and Scandola (p-value < 0.01) and the Calanques and Scandola (p-value < 0.001) populations, but no significant differences were found between Medes and Calanques (p-value > 0.05) (Fig. 4a). When considering the results of the one-way PERMANOVA, the Population factor was significant (p-value < 0.001) (Supplementary Table S2), and the pairwise post-hoc results revealed that many pairs of populations display significant differences in the response to thermal stress, most of them involving Scandola (Supplementary Table S3). However, several pairs of populations from different localities and depths did not exhibit significant differences (p-value > 0.05, Supplementary Table S3, Fig. S1). For example, no significant differences were found between the pairs Medes shallow - Calanques 1 shallow and Medes deep – Scandola deep (Supplementary Table S3, Fig. S1). Moreover, no significant differences were found between depths at any of the three localities (Supplementary Table S3, Fig. S1).

#### Importance of biological processes

Population genetic structure. Genetic characteristics of the populations including the level of null alleles, heterozygosity and *f* computations are presented on the Supplementary Results S1. The global θ was 0.075 (95% CI: 0.044–0.134), and the pairwise values and 95% CI for θ ranged from 0.028 (95% CI: 0.010–0.041) for Calanques 3 shallow vs. Calanques 1 shallow to 0.137 (95% CI: 0.052–0.230) for Calanques 1 shallow vs. Scandola. The population-specific *F*_*ST*_ values varied from 0.052 (95% CI: 0.033–0.073) for Calanques 2 shallow to 0.090 (95% CI: 0.058–0.127) for Calanques 1 shallow (Table 1).

**Table 1:** Pairwise *P*_*ST*_ and *F*_*ST*_ results.

Pairs	*P*_*ST*_	*P*_*ST*_ CI	*F*_*ST*_	*F*_*ST*_ CI
C1-sh vs. C2-sh	0	(0–0.135)	0.028	(0.010–0.041)
C1-sh vs. C3-sh	0.065	(0–0.314)	0.049	(0.026–0.0627)
C2-sh vs. C3-sh	0.012	(0–0.131)	0.041	(0.020–0.057)
C1-sh vs. C1-d	0.000	(0–0.08)	0.045	(0.026–0.0761)
C2-sh vs. C1-d	0	(0–0.095)	0.033	(0.011–0.055)
C3-sh vs. C1-d	0.028	(0–0.210)	0.045	(0.017–0.074)
C1-sh vs. M	0.012	(0–0.102)	0.131	(0.021–0.277)
C1-sh vs. S	0.069	(0–0.262)	0.137	(0.052–0.230)
C2-sh vs. M	0.019	(0–0.106)	0.094	(0.021–0.193)
C2-sh vs. S	0.128	(0.027–0.385)	0.116	(0.062–0.161)
C3-sh vs. M	0.072	(0–0.215)	0.126	(0.036–0.254)
C3-sh vs. S	0.235	(0–0.521)	0.127	(0.060–0.207)
C1-d vs. M	0.004	(0–0.09)	0.085	(0.028–0.170)
C1-d vs. S	0.076	(0–0.265)	0.095	(0.048–0.137)
M vs. S	0.028	(0–0.124)	0.053	(0.020–0.076)

Pairwise *P*_*ST*_ and *F*_*ST*_ results with their corresponding 95% confidence intervals (CI). C1-sh, C2-sh and C3-sh: Calanques 1, 2 and 3 shallow, C-d: Calanques 1 deep, M: Medes, S: Scandola.

**Figure 5:**
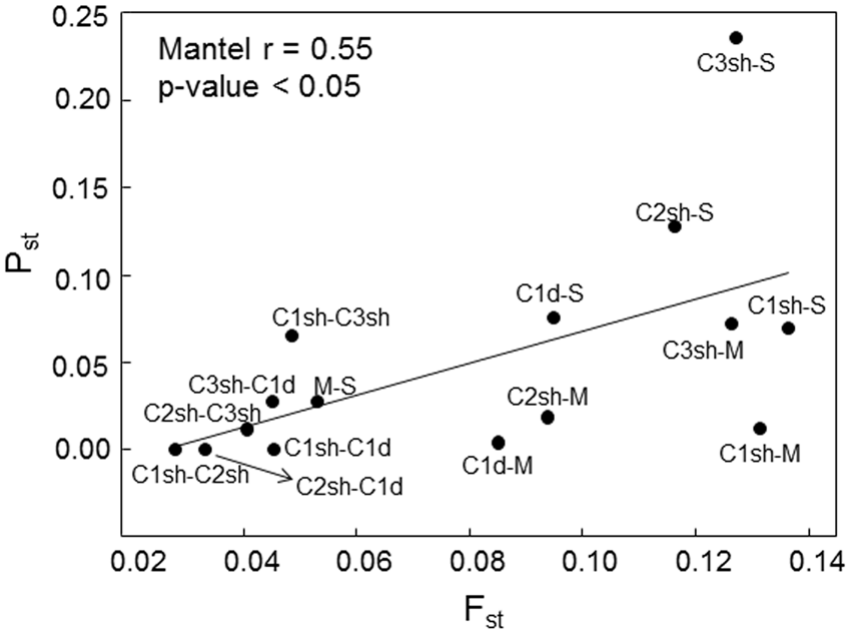
Pairwise *P*_*ST*_ vs. pairwise *F*_*ST*_. Relation between *P*_*ST*_ and *F*_*ST*_. Results of a Mantel test with the corresponding p-value and the regression line are shown. M: Medes; C1sh, C2sh and C3sh: Calanques 1, 2 and 3 shallow respectively; C1d: Calanques 1 deep; S: Scandola.

Relationship between neutral genetic structure and phenotypic response. The global *P*_*ST*_ under the null assumption (*c/h* ^*2*^ = 1) was 0.25 (95% CI: 0.007–0.436), and the pairwise *P*_*ST*_ values ranged from 0 for Calanques 3 shallow vs. Calanques 1 shallow, Calanques 3 shallow vs. Calanques 1 deep and Calanques 1shallow vs. Calanques 1 deep (95% CI were 0–0.135, 0–0.095 and 0–0.08, respectively) to 0.235 (95% CI: 0–0.521) for Calanques 2 shallow vs. Scandola (Table 1). Pairwise *P*_*ST*_ were positively and significantly correlated to pairwise *F*_*ST*_ (Mantel r coefficient = 0.55, p value < 0.05, Fig. 5), but the global *P*_*ST*_ was not significantly different from the global *F*_*ST*_ based on the respective 95% CIs. In all of the pairwise *P*_*ST*_ and *F*_*ST*_ comparisons, the 95% CIs overlapped, suggesting a lack of significant differences between the two measures (Table 1). Therefore, the patterns of neutral genetic and phenotypic differentiations were identical and likely driven by the same process.

**Figure 6:**
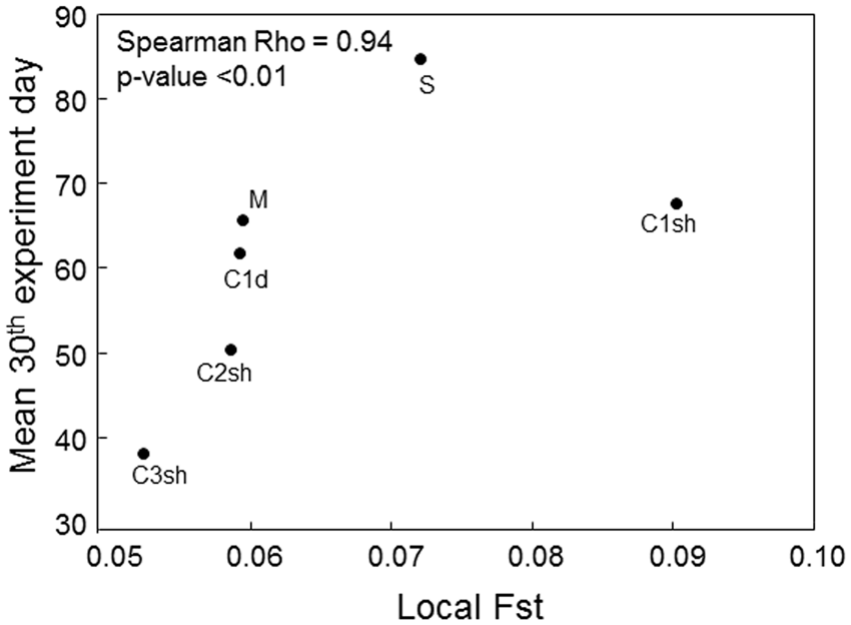
Affectation vs. local *F*_*ST*_. Relation between affectation (mean percentage of necrosis on the last day of the experiment) and local *F*_*ST*_. The points and their labels are shown. The Spearman Rho statistic and its corresponding p-value are also presented. M: Medes; C1sh, C2sh and C3sh: Calanques1, 2 and 3 shallow; C1d: Calanques 1 deep; S: Scandola.

A positive and significant correlation was obtained between the population-specific *F*_*ST*_ and the mean percentage of necrosis at the last day of the experiment (Spearman Rho = 0.94; p-value < 0.01). Accordingly, genetically isolated populations seemed to be more impacted by thermal stress (Fig. 6).

## Discussion

In this study, we conducted a comprehensive characterization of the response to thermal stress of *Paramuricea clavata* populations thriving in distinct temperature regimes, i.e. from localities separated by hundreds of kilometers and from different depths. Through a common garden thermotolerance experiment, we demonstrated the limited influence of local temperature regimes and suggested that genetic drift may hinder the impact of divergent selection and modulate the response of populations to thermal stress.

Our study indicated that 25 °C is a critical temperature for *P. clavata* because all of the analyzed populations exhibited the first signs of necrosis after one week of exposure. When populations were exposed to higher temperatures (≥26 °C), colonies were affected by necrosis from the first day, and most of them suffered extensive damage in less than 5 days. Other experimental studies also indicated that similar short-term exposure to 25 °C was critical for triggering necrosis in *P. clavata* ^38, 39^. At lower temperatures (23–24 °C), 49 days of exposure were required for the first signs of necrosis to be observed^35^. Overall, these results are consistent with *in-situ* temperature surveys conducted during the mortalities events in 1999 and 2003 in different areas of the NWM^7, 37^. They are also supported by previous experimental studies focused on other anthozoans affected during these events. Indeed, in experimental conditions, 25 °C was identified as critical temperature for aposymbiotic species such as the red coral *Corallium rubrum* and the yellow gorgonian *Eunicella cavolini* ^31, 32^.

Since temperatures higher than 25 °C are rarely observed in the studied localities^36, 37^, we suggest that the shallow (marginal) populations of *P. clavata* are living near their upper thermal limit. This hypothesis is strengthened by the fact that Mediterranean gorgonians, in general, and *P. clavata*, in particular, shift their distribution to greater depths in warmer areas^40, 41^. Specifically, Linares *et al*.^42^ noted this upper depth limit distribution on the latitudinal scale with populations inhabiting from 10–15 m at 42° 22′N and from 30–35 m at 37° 38′N associated to the changes in the temperature conditions. Coupling this information on geographic and bathymetric species distributions to projected ocean warming and to population response variability would enable the identification of areas that are particularly vulnerable to climate change^43–45^ and the design of efficient specific conservation measures^45^.

The populations of *P. clavata* dwelling in warm conditions did not exhibit a higher resistance to thermal stress than those found in colder conditions. This result contrasts with previous studies conducted in various marine species suggesting that populations from warm habitats were more tolerant to thermal stress than populations from cold habitats (e.g. refs 15, 17, 46 and 47). In these cases, local adaptation and acclimatization processes related to local temperature conditions were used to explain the differential responses to thermal stress. Our study takes the opposing view of these results since the differential responses reported here seems only slightly related to local thermal conditions. While the observed positive correlation between phenotypic divergence (*P*_*ST*_) and the neutral genetic divergence among populations (*F*_*ST*_) may be due alternatively to selective or neutral processes^48^, the low and mostly similar pairwise *P*_*ST*_s and *F*_*ST*_s values combined to the positive correlation between the population-specific *F*_*ST*_ and the level of necrosis at the end of the experiment support the influence of genetic drift. Accordingly, we hypothesize that the differential responses to thermal stress in *P. clavata* is mainly driven by genetic drift, a stochastic and neutral evolutionary processes.

In line with this hypothesis, recent studies suggest that genetic drift may have a dominant impact on phenotypic differentiation in Mediterranean octocorals. The level of genetic drift was suggested as a limiting factor in the adaptation of red coral populations to their local environments^29^ while, a genetic survey of *P. clavata* populations recently impacted by a mortality event demonstrated a positive link between the extent of genetic drift and the level of tissue necrosis^49^. Moreover, this hypothesis is consistent with model predictions^50, 51^ and the results of a meta-analysis^52^. Indeed, the influence of genetic drift on local adaptation is expected to be stronger in species with restricted connectivity and low effective population size^50–52^ as reported in *P. clavata* ^53, 54^. Considering these life history traits are shared by other Mediterranean anthozoans^55, 56^, the lack of local adaptation to thermal conditions may be widespread in these species questioning their abilities to deal with on-going climate change.

One should highlight the limitations linked to the *P*_*ST*_ computations^57–59^. Genomic studies^60^ are thus needed to further decipher the respective roles of selective and neutral processes on the evolution of *P. clavata* and similar anthozoans in the environmental shift.

Understanding the response of populations to climate change is a central topic in conservation biology as a way to better assess species viability in the currently changing ocean. Our study highlights the potential impact of genetic drift in the response to thermal stress, which complements the picture of previously identified modulating factors (e.g., physiological status^35^, thermodependent pathogens^61^, sex^53^). Taking this information into consideration should improve our capacity to more accurately characterize the vulnerability of marine coastal ecosystems. In this sense, we addressed the importance of the assessment of genetic drift on populations of *P. clavata* at different spatial scales, which is especially urgent in areas where warming is expected to reach critical temperatures, such as the North Western Mediterranean^62^.

This approach will allow the more effective design of conservation measures encompassing a comprehensive range of spatial scales to preserve the adaptive potential of marine populations: from the design of MPA networks to specific restoration actions at the population level in view to promote the connectivity and controlling density erosion of populations. Including this information in management plans will contribute to the conservation rich Mediterranean marine biodiversity, in the face of expected warming^62^.

## Methods

### Model species

The red gorgonian *Paramuricea clavata* (Risso, 1826) (Cnidaria, Anthozoa, Octocorallia) was the model species chosen for this study. This species is considered a key species of Mediterranean coralligenous assemblages and is also one of the species that has been most affected by recent mass mortality events^7, 33, 34^.

### Study localities and temperature regimes

We considered three localities separated by hundreds of kilometers within the NWM region: the Medes Islands (Catalonia, NE Spain), Calanques (Provence, France) and Scandola (NW Corsica, France) (Fig. 1a). These three localities were chosen based on 1) their contrasted temperature regimes and features of thermal anomalies (Fig. 2, subsection Temperature regimes of the study localities of the Results Section^36, 37^), 2) their differential degrees of impact during the recent regional mass mortality events^7, 36, 37^ and 3) their association with distinct genetic clusters^54^.

At each locality, two populations dwelling at shallow (20 m) and deep (40 m) depths, and thus separated by tens of meters, were sampled (Fig. 1b–d). In Calanques, two additional shallow (20 m) populations separated between 4 to 6 km were sampled (Fig. 1c). Throughout the text, the studied populations are referred as Medes shallow, Medes deep, Calanques 1 shallow, Calanques 1 deep, Calanques 2 shallow, Calanques 3 shallow, Scandola shallow and Scandola deep.

Temperature regimes of the studied localities were characterized in previous studies^36, 37^. Here we provide a description of the main attributes of thermal regimes of the different localities and depths where the populations were sampled through the analysis of hourly temperature time series provided by TMEDNet (http://www.t-mednet.org). The longest possible period for which data was available at the 3 sites for a given depth were considered, i.e. 11 years (2004–2014) for 20 m depth and 8 years (2004–2011) for 40 m depth. From these long term series, average annual cycle and statistics on the distribution of hourly records were calculated. Annual cycle is based on daily average temperatures. For each julian day, mean ± SD were calculated. Boxplots were used to summarize information on central tendency, dispersion and extreme values of hourly temperature records: first, to characterize conditions during warm months, from July to end of October (hereafter warm period); then, to characterize distribution of extreme hot temperatures during the warm period (from 90^th^ percentile).

### Experimental setting and biological material

We carried out a common garden experiment (i.e. individuals from different origins are transplanted to a new habitat where they are submitted to a common biotic -e.g. high predation- or abiotic -e.g. high temperature- environmental factor^18^) in aquaria with the selected *Paramuricea clavata* populations. We performed four independent common garden thermotolerance experiments in aquaria, considering for each, four different temperatures respectively: 25 °C, 26 °C, 27 °C and 28 °C (see Experimental Design subsection). All the experiments were conducted between July and November of 2009 with apical tips (between 5 and 7 cm in length) of healthy, mature red gorgonian colonies (20–40 cm in height^63^). Apical tips (hereafter colonies) were randomly sampled in the study localities by SCUBA diving after 2009 spawning occurred. All the colonies were sampled in June 2009 except for those of the 28 °C experiment which were sampled in November 2009 (see Supplementary Table S4).

Immediately after sampling, each colony was assembled on experimental plates and placed in coolers equipped with air pumps (battery powered) to be transported to experimental aquarium facilities at the Institute of Marine Sciences-CSIC (Barcelona, Spain). Experimental plates were built up by three PVC rectangular pieces (5 × 30 cm in size) and two rubber layers. Each PVC piece had 10 holes (1 cm in diameter) to place the colonies. The colonies were fixed in the experimental plates without using any putty, instead the colonies were hold mechanically thanks to the perpendicular cuts previously done in the rubber layers (one cut in each layer) (see Fig. S2).

**Figure 7:**
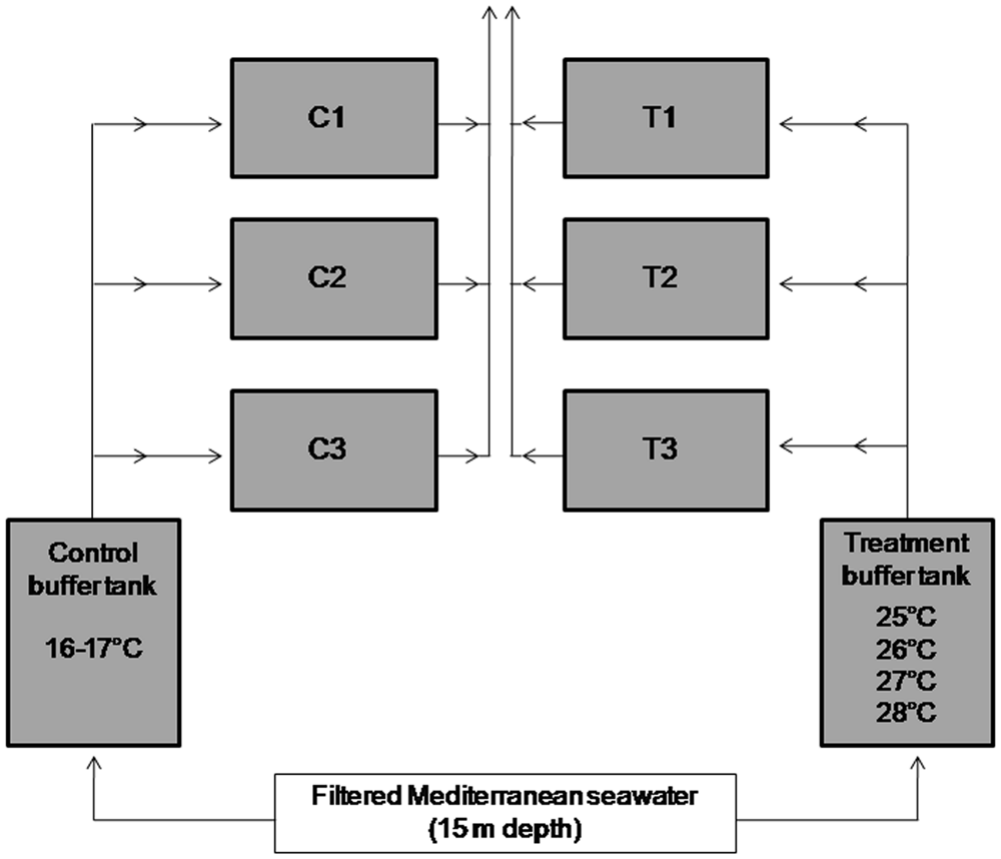
Experimental setting. Experimental setting of all the performed experiments. Control and Treatment sets where colonies were placed were composed by three replicates (C1, C2 and C3, and T1, T2 and T3 respectively) plus one large buffer tank which was supplied with filtered Mediterranean seawater and from there, water was pumped continuously into the experimental tanks. Buffer tanks were used to control the temperature of experimental tanks.

The maximum transportation time was of 36 h for the colonies collected in Scandola (Corsica, France) while the minimum time was of several hours for the colonies collected in Medes (Catalonia, Spain). The temperature in the coolers was monitored regularly and seawater ice packs were used when necessary to maintain the water temperature between 15–18 °C. Once in the aquarium, colonies were maintained at 16–17 °C until the experiments started (Supplementary Methods S1). At the moment of starting with the experiments 100% of the colonies were healthy, presenting expanded polyps during feeding events and no tissue necrosis. All of the experiments used the same setting with two aquarium sets: Control and Treatment. Both Control and Treatment sets were composed by three replicates (three tanks of 105 l each), where colonies were placed, plus one large buffer tank (≈ 100 l) (Fig. 7). The buffer tanks were supplied with filtered Mediterranean seawater (pumped at 15 m depth) and from there, water was pumped continuously into the experimental tanks. Buffer tanks were used to control the temperature of experimental tanks (Fig. 7).

In the Control, seawater was maintained at 16 to 17 °C. We decided to set this temperature as control temperature because it corresponds to low temperatures during the summer period in all localities and it was also the Control temperature used in previous experimental studies with corals of the same study region^31, 38, 64^. In Treatment sets, seawater was heated (see the Experimental design subsection) in the buffer tank with submersible resistance heaters and was regulated by temperature controllers (Aqua Medic T controller). All the tanks were equipped with submersible pumps to facilitate water circulation within the tanks. Temperature was registered in the Control and Treatment aquariums with Tidbit Stowaway autonomous temperature sensors every half an hour.

Depending on the experiment, 15 to 30 colonies were used per population and per Control/Treatment set (Table S4). The experimental sets functioned as an open system.

To check the physiological status of the colonies during the experiments, the proxy of the surface area (cm^2^/branch linear cm^35^) was used. Overall the results showed that colonies placed in the Control followed similar variations in surface area than those in the field (Supplementary Methods S1).

### Colonies feeding

In the Control and Treatment sets, colonies were fed two days per week, one day with nauplii of *Artemia salina* (1076.7 ± 156.7 org/l) and the other with phytoplankton diatom *Tetraselmis chuii* (chl-*a* = 3.3 ± 0.4 ug/l) (these concentrations correspond to those of each experimental tank). After the addition of food, the tanks were maintained in closed system mode for 5 h. During this period, water circulation within tanks was supplied by submersible pumps.

The strategy of supplying pulses of food instead of a constant supply was based on the results of *in situ* studies of food abundance and prey capture at short time scales in benthic suspension feeders^65^. The results of sampling food abundance near the organisms and their gut contents demonstrated that organisms commonly acquire available resources in short time pulses. There was estimated that these pulses of food abundance occurred between 2 and 3 times per week on average^65^, so that was simulated in the performed experiments. The period of 5 h was chosen on the basis of the duration of the pulses of food abundance in the field^65^ and because previous experimental studies with *P. clavata* indicated that 5 h is an appropriate period, after which food abundance reaches similar levels prior to starting the feeding^66^. Furthermore, we measured food concentrations before, during and after the feeding events, and we arrived to the same results (results not shown). To obtain an estimate of food concentration during the experiments, we measured the particulate organic carbon (POC) concentration which is the most integrative descriptor of food conditions^66^. The POC concentration throughout the experimental treatment was 323 ± 50 ug C/l. POC values that characterize coastal waters in the NW Mediterranean are on the order 300 ug C/l^67, 68^, hence, provided food concentrations were similar to those observed in the summer period in the field.

Overall, the frequency and amount of food together with the maintenance of the system in a close mode after feeding, guaranteed that colonies were supplied with a surplus of food as observed in previous experimental studies^66^.

### Response variable

For all of the experiments, the response variable was the level of tissue necrosis, which was visually monitored daily and assessed as the percentage of the colony exhibiting dead tissue (from clear grey to black) or denuded axis (see Fig. S3). From these observations, the percentage of affected colonies (i.e., the proportion of colonies displaying tissue necrosis >0%) and mean necrosis values per day were computed to assess the response to thermal stress.

### Experimental design

The common garden experiments were designed to assess the thermotolerance thresholds of *Paramuricea clavata* and to evaluate the importance of environmental and neutral genetic factors in the response of *P. clavata* populations to thermal stress. Four experiments considering the following temperatures were carried out: 25, 26, 27 and 28 °C (see next subsection).

#### Assessing the thermotolerance thresholds of Paramuricea clavata

We carried out four experiments to evaluate the thermotolerance thresholds of *P. clavata*. The first experiment consisted of a stepwise temperature augmentation from 18 °C to 22 °C by 2 °C per week and then by 1 °C per week until 25 °C, which was identified as the critical temperature for *P. clavata* (see Results). Once this temperature was reached, the conditions were kept constant for 30 days (until at least 95% of the colonies were affected by tissue necrosis).

To explore the response to thermal stress above this temperature, we performed three additional experiments with constant temperature of 26, 27 and 28 °C. These temperatures were attained after one day of gradual augmentation from 18 °C to the treatment temperature, and the experiments were conducted until at least 95% of the colonies were affected by necrosis. The 26, 27 and 28 °C experiments had a total duration of 13, 6 and 4 days respectively (Table S4). The 25, 26 and 27 °C experiments were carried out using the eight populations while the 28 °C experiment was only performed with two populations (Medes shallow and deep) due to logistical constraints (Table S4).

For each population and experiment, we reported the daily percentage of affected colonies. Then, to characterize the thermotolerance features of *P. clavata*, for each experiment, we calculated the mean (±SE) daily percentage of affected colonies across populations.

#### Exploring the factors and processes modulating the response of populations to thermal stress

To explore the influence of environmental and genetic factors on the response of *P. clavata* populations, we considered the common garden experiment at 25 °C because this temperature was identified as critical for *P. clavata* (see Results).

Environmental factors. We postulated that populations from depths/localities with colder thermal regimes will be more affected by thermal stress than populations subject warmer thermal regimes. In particular, and bearing in mind the temperature regimes of the selected localities and depths^36, 37^ (see Results section), we expected shallow populations to be less affected by thermal stress than those in deeper water. Regarding the localities, the Scandola and Calanques populations should be the least and most affected, respectively, while the Medes population should present an intermediate response. To analyze these hypotheses, we focused on the response to temperature of six populations coming from three localities and two depths with contrasting thermal regimes: Medes shallow and deep, Calanques 1 shallow and deep and Scandola shallow and deep^36, 37^. The two populations from Calanques 2 and Calanques 3 shallow were not included to maintain a balanced design in the analysis (see below).

To assess statistical differences in tissue necrosis in the Treatment between localities and depths, we performed a two-way multivariate PERMANOVA^69, 70^ with Locality (three levels) and Depth (two levels) as fixed factors. A multivariate approach, taking into account tissue necrosis from three days covering the whole experiment (the 10^th^, 20^th^ and 30^th^ days) as the multivariate dependent variable was considered^69^. Post-hoc pair-wise PERMANOVA tests were performed to identify significant differences between pairs of localities. Complementarily, we ran a second PERMANOVA taking into account the six populations but accounting for only one factor (Population) that was considered fixed. The aim of this one-way analysis was to obtain pair-wise (through post-hoc pair-wise tests) results for all of the populations without restricting comparisons within depths or localities. The same multivariate approach of the previous experiment was considered. The colonies from the Control sets were not considered in these analyses because they remained healthy (no tissue necrosis) until the end of the experiment. For all of the tests, 9999 permutations were achieved. PERMANOVA analyses were performed in PRIMER v6^71^ in conjunction with the Windows PERMANOVA + module^72^. For a more detailed description of the statistical analyses, we refer to Supplementary Methods S2.

Biological processes. Phenotypic divergence between populations, such as the differential response to thermal stress, results from the interaction between neutral and adaptive processes^73, 74^, and one way to characterize this interaction is to estimate and compare the neutral genetic divergence to the phenotypic divergence for the considered trait^58^. Through this characterization, we aimed to obtain the first insight into the role of population genetic features on the observed differential responses to thermal stress.

Neutral genetic divergence: Before initiating the experiments, a small tissue sample was taken from each apical tip and stored in 96% ethanol and at −20 °C for subsequent genetic analysis. Two hundred and forty colonies belonging to the eight populations were genotyped using six microsatellite loci (Parcla-09, Parcla-10, Parcla-12, Parcla-14, Parcla-17 and Par-d) following^54^ DNA extraction and genotyping protocols are detailed in Supplementary Methods S3.

As a preliminary step, we identified the different genetic pools (i.e., genetically distinct populations) within our dataset by conducting exact tests of the overall genotypic differentiation among populations and between all pairs of populations in GENEPOP^75^ using the default parameters. These analyses showed that the two populations from Medes (shallow and deep) and the two populations from Scandola (shallow and deep) were not significantly genetically different (p-values > 0.001). Accordingly, we grouped Medes shallow and Medes deep in the Medes genetic pool and Scandola shallow and Scandola deep in the Scandola genetic pool, so the following analyses were conducted considering six different genetic pools (Calanques 1 shallow, Calanques 1 deep, Calanques 2 shallow, Calanques 3 shallow, Medes and Scandola).

We estimated the neutral genetic divergence and respective 95% confidence intervals (95% CIs) by computing the global and pairwise differentiation between the six genetic pools using *θ*, the Weir & Cockerham estimator of *F*_*ST*_^76^ in FREENA^77^. We also calculated the population-specific *F*_*ST*_ as implemented in GESTE^78^ for the six genetic pools. This method measures how genetically distinct a population is compared to all of the populations combined and provides insight into the relative impact of genetic drift and gene flow on the differentiation of each population^78^.

Phenotypic divergence: The global and pairwise phenotypic divergences were estimated between the six genetic pools using *P*_*ST*_^58, 79, 80^, which is defined as *P*_*ST*_ = *cσ* ^*2*^_*GB*_/(*cσ* ^*2*^_*GB*_ + *2h* ^*2*^ *σ* ^*2*^_*GW*_), where *σ* ^*2*^_*GB*_ and *σ* ^*2*^_*GW*_ represent the among-sample (in this context one sample refers to one genetic pool) and the within-sample variance components for the considered phenotypic trait, respectively; *h* ^*2*^ represents the assumed additive genetic proportion of the differences between individuals within samples (“narrow-sense heritability”); and *c* represents the proportion of the total variance presumed to occur because of additive genetic effects across samples^81^. We considered the null assumption that the genetic architecture of the trait (i.e., the level of tissue necrosis) remained equal across samples with *c/h* ^*2*^ = 1^80, 81^. Among- and within-sample variances were computed based on PERMANOVAs, which were conducted considering “the level of tissue necrosis of the 10^th^, 20^th^ and 30^th^ experiment day” of the common garden experiment as a multivariate dependent variable and “genetic pool” as the only random factor (with six levels: Medes, Calanques 1 shallow, Calanques 1 deep, Calanques 2 shallow, Calanques 3 shallows and Scandola).

Variances were estimated by extracting the within-sample and between-sample mean squares (*MS*_*B*_ and *MS*_*W*_, respectively) from the PERMANOVA output. The within-sample variance (*σ* ^*2*^_*GW*_) was directly estimated based on the within-sample *MS*_*w*_. The between-sample variance (*σ* ^*2*^_*GB*_) was estimated as *σ* ^*2*^_*GB*_ = (*MS*_*B*_ − *MS*_*W*_)/*n*_*0*_ with *n*_*0*_ being a weighted average of the sample size for each comparison:

$${n}_{0}=\frac{1}{a-1}(\sum ^{n}{n}_{i}-\frac{\sum _{a}{{n}_{i}}^{2}}{\sum _{a}{n}_{i}})$$

where *a* is the number of samples to be compared, and *n*_*i*_ is the number of individuals in the *i* ^th^ sample^82, 83^. Negative variance values were set to zero^84^.

We obtained each *P*_*ST*_ by calculating 1000 *P*_*ST*_ from 1000 PERMANOVAs considering 1000 bootstrap samples of the multivariate dependent variable. This allowed us to estimate the final *P*_*ST*_ values as the median values over the 1000 permutations along with the 95% CI, which corresponds to the 25^th^ and 975^th^ values of the sorted *P*_*ST*_ respectively. Finally, we considered 100 permutations for each PERMANOVA.

Comparison between genetic and phenotypic divergences: We conducted a Mantel test (n = 1000) to analyze the relationship between pairwise genetic (*F*_*ST*_) and phenotypic (*P*_*ST*_) estimates of divergence. A significant relationship between the two estimates of divergence suggests that the pattern of phenotypic divergence is mainly explained by genetic drift^58, 85^ or, alternatively, that isolation by adaptation occurs with divergent selection driving the evolution of the phenotypic trait and the differentiation at neutral loci^86, 87^. To disentangle the two hypotheses, we then compared the *P*_*ST*_ (under *c/h* ^*2*^ = 1) and *F*_*ST*_ estimates for each population pair. If divergent selection is involved in the observed patterns, we expect the *P*_*ST*_ estimates to be significantly higher than *F*_*ST*_^58^. The comparison of the estimators was based on their 95% CIs.

Using a non-parametric Spearman correlation, we further examined the impact of genetic differentiation on the response to thermal stress by testing for the relationship between the population-specific *F*_*ST*_ and the level of necrosis of each population observed on the last day of the experiment. A positive correlation is expected when isolated populations, which are subjected to a stronger genetic drift effect, are more impacted by thermal stress.

The PERMANOVA (adonis function), *P*_*ST*_ computations (personal scripts available in Supplementary Methods S4), Mantel test (mantel function) and Spearman correlation (cor.test function) were implemented in R version 3.2.1 (http://cran.r-project.org).

## Electronic supplementary material

Supplementary information
