# Supplementary material for: Regional and local environmental conditions do not shape the response to warming of a marine habitat-forming species
Source: Sci Rep. 2017 Jul 11;7:5069. doi: 10.1038/s41598-017-05220-4 (PMC5505982; doi:10.1038/s41598-017-05220-4)
Supplement: Supplementary file 1 — Supplementary information [file 41598_2017_5220_MOESM1_ESM.pdf]

SUPPLEMENTARY INFORMATION (displayed in the same order that appears in the main text)

Regional and local environmental conditions do not shape the response to warming of a marine habitat-forming species

Crisci C[1*], Ledoux J-B[2,3],Mokhtar- Jamaï K[4], Bally M[5], Bensoussan N[6], Aurelle D[4], Cebrian E[7,8], Coma R[7], Féral J-P[4], La Rivière M[5], Linares C[9], López-Sendino P[3], Marschal C[4],Ribes M[3], Teixidó N[10], Zuberer F[11], Garrabou J[3,5]

Table S1. Two-way PERMANOVA results.Two-way PERMANOVA results from the common garden experiment considering the factors Locality and Depth and their interaction (Lo x De). Six populations were considered: Medes shallow and deep, Calanques 1 shallow and deep, Scandola shallow and deep. Df: degrees of freedom, SS: sum of squares, MS: mean squares, Pseudo-F: pseudo F statistic, P(perm): PERMANOVA p-value.

Factor	Factor levels	Df	SS	MS	Pseudo-F	P(perm)
Locality (Fixed)	Medes, Calanques 1, Scandola	2	50045	25023	7.9252	0.0001
Depth (Fixed)	20 m, 40 m	1	10077	10077	3.1916	0.0495
Lo x De		2	8268.4	4134.2	1.3094	0.2588
Res		174	549380	3157.3		
Total		179	617770			

Table S2. One-way PERMANOVA results. One-way PERMANOVA results from the common garden experiment considering the factor Population as a fixed factor. The same six populations of the two-way PERMANOVA were considered. Df: degrees of freedom, SS: sum of squares, MS: mean squares, Pseudo-F: pseudo F statistic, P(perm): PERMANOVA p-value, M-sh: Medes shallow, M-d: Medes deep, C1-sh: Calanques 1 shallow, C1-d: Calanques 1 deep, S-sh: Scandola shallow, S-d: Scandola deep.

**Factor**	**Factor levels**	**Df**	**SS**	**MS**	**Pseudo-F**	**P(perm)**
Population (Fixed)	M-sh, M-d, C1-sh, C1-d, S-sh, S-d	5	68391	13678	4.3322	0.0002
Res		174	549380	3157.3		
Total		179	617770			

Table S3.Post-hoc pairwise results of the one-way PERMANOVA. Significance of the results of the post-hoc pairwise one-way PERMANOVA. ***: p-value<0.001, **: p-value<0.01, *: p-value<0.05, ns: not significant. Dark grey cells indicate comparisons between populations at the same locality. M-sh: Medes shallow, M-d: Medes deep, C1-sh: Calanques 1 shallow, C1-d: Calanques 1 deep, S-sh: Scandola shallow, S-d: Scandola deep.

	**M-sh**	**M-d**	**C1-sh**	**C1-d**	**S-sh**	**S-d**
**M-sh**						
**M-d**	ns					
**C1-sh**	ns	*				
**C1-d**	ns	*	ns			
**S-sh**	*	Ns	*	*		
**S-d**	***	Ns	***	***	ns	

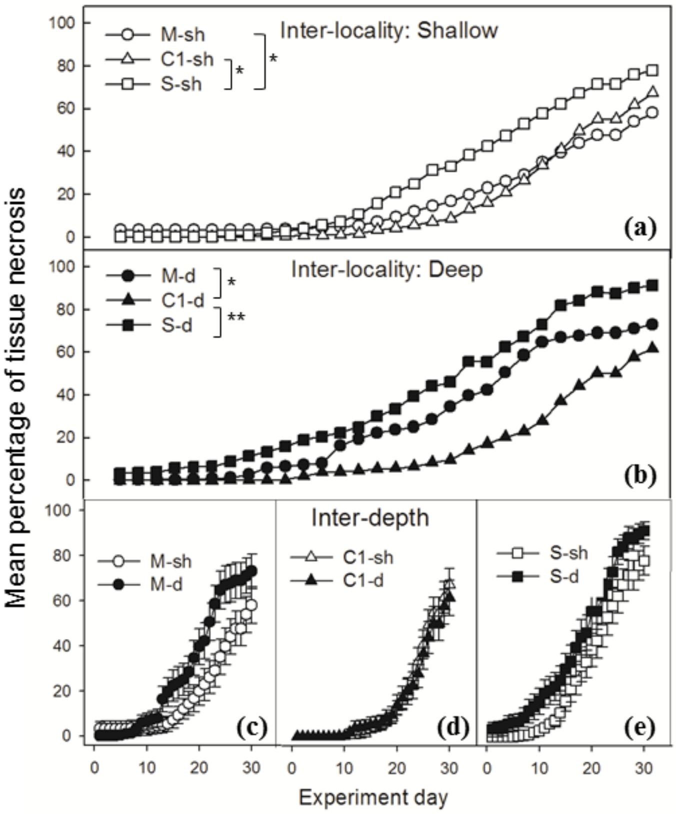

Figure S1. Alternative representation of tissue necrosis of the studied populations. Mean percentage of tissue necrosis of populations of the three study localities at 20 (a) and 40 m depth (b), and of both depths inside each locality (c,d,e) during the 25ºC common garden experiment. Following the test design of the two-way PERMANOVA analysis, only the Calanques1 shallow and Calanques 1 deep populations of theCalanques locality were considered in this figure. The level of significance of the differences between pairs of localities and depths (as a result of the two-way PERMANOVA post-hoc pairwise test) is indicated as follows: **: p-value<0.01, *: p-value<0.05. The absence of indications between pairs denotes the absence of significant differences. M-sh: Medes shallow, M-d: Medes deep, C1-sh: Calanques 1 shallow, C1-d: Calanques1 deep, S-sh: Scandola shallow, S-d: Scandola deep.

Supplementary ResultsS1. Genetic characteristics of the populations.

Large allele dropout and scoring errors due to stuttering were not observed in the dataset. All loci were polymorphic in all the samples. The frequencies of null alleles (r) ranged from 0.01 for Medes shallow to 0.03 for Calanques1-shallow, Calanques1deep, Calanques2shallow and Scandola deep with a mean value of 0.02 (+/- 0.04) over all populations. No significant linkage disequilibrium was observed overall samples or within each sample. The mean observed heterozygosity (H_o) was 0.76 (+/- 0.31) and ranged from 0.7 for Calanques 1 shallow to 0.79 for Calanques 1 deep. The gene diversity (H_e) varied from 0.68 for Calanques 1 shallow to 0.78 for Medes deep and Calanques 1 deep with a mean value of 0.74 (+/- 0.03) over all populations. Significant heterozygote deficiencies were observed in three samples namely Medes deep, Calanques 1 shallow and Calanques 2 deep and the f values ranged from 0 for Medes shallow to 0.15 for Calanques 1 deep.

Table S4. Principal features of the experiments. The number of colonies per population where equally distributed among the three control/stress replicate tanks (e.g., in the 18-25ºC ladder experiment, there were 10 colonies from each population per tank). For the common garden experiment, we took advantage of the 18-25ºC ladder experiment but only considered exposure to 25ºC. M-sh: Medes shallow, M-d: Medes deep, C1-sh, C2-sh, C3-sh: Calanques1, 2 and 3 shallow, C1-d: Calanques1 deep, S-sh: Scandola shallow, S-d: Scandola deep.

	**Temperature (ºC)**	**Nº pops. (localities /depths)**	**Populations**	**Nº of colonies per population (control/stress)**	**Total nº of colonies**	**Duration (days)**	**Starting date**
Thermotolerance features	18-25 (ladder)	8 (3/2)	M-sh, M-d, C1-sh, C1-d, C2-sh, C3-sh, S-sh, S-d	30/30	480	63	01/07/2009
	26 (constant)	8 (3/2)	M-sh, M-d, C1-sh, C1-d, C2-sh, C3-sh, S-sh, S-d	15/15	240	13	02/09/2009
	27 (constant)	8 (3/2)	M-sh, M-d, C1-sh, C1-d, C2-sh, C3-sh, S-sh, S-d	15/15	240	6	04/11/2009
	28 (constant)	2 (1/2)	M-sh, M-d	30/30	120	4	10/11/2009
Common garden experiment	25 (constant)	8 (3/2)	M-sh, M-d, C1-sh, C1-d, C2-sh, C3-sh, S-sh, S-d	30/30	480	30	02/08/2009

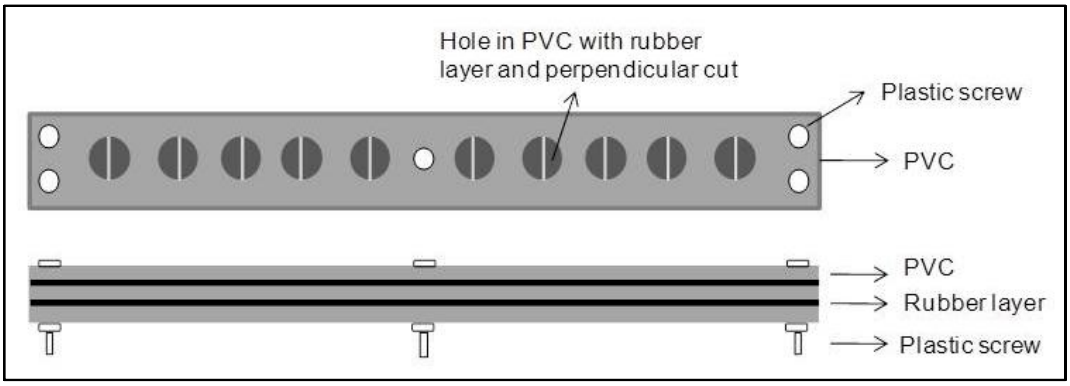

Figure S2. Experimental plates. Top and side view of experimental PVC plates were colonies were placed.

Supplementary Methods S1.Considerations on the time that colonies were placed in aquaria before starting the experiments and on energy reserves.

Time that colonies were placed in aquaria

Upon on the arrival of the samples were placed in the aquarium at 16-18ºC (temperature used in the control treatment) during 1 to several weeks. At our knowledge there is no information on the time required to adjust to the change in environment. However, since all samples were sampled and transported using identical treatment procedures we contend that the results were due to the populations' capacity to cope with thermal stress. During the experiment we conducted parallel tests to check the potential effect of acclimation period using different time from 1 day to 40days. In these settings the final outcomes in the response to the thermal stress were not significantly different. Besides other experiments carried with different red gorgonian *Paramuricea clavata* populations (see for instance [1–3]) showed that 25ºC was the temperature threshold regardless the different timings used before and during the warming up period. Finally, the observed status of all control colonies at the end of the experiments (polyp activity and no tissue

necrosis after feeding events) indicates that there was not effect of the time that colonies spent in aquaria before starting with the experiments.

Energy reserves

In order to determine whether or not the experimental conditions were suitable for the populations, we quantified the changes in energy reserves of the Control colonies over the experimentation as well as over sampled colonies in the field. As a proxy of energy reserves, we quantified the surface area (cm^2/branch linear cm). The measure on surface areas was carried out using the NIH Image software. We compared the surface area (cm^2/branch linear cm) of Control colonies at the beginning and at the end of the ladder experiment (see Table S4) conducted during the summer period, with that of colonies collected in the field during the fall (November 8). Overall there were significant differences in surface area between the three groups of colonies ($F_{2,27}$=7.006, p-value=0.0035). The differences where due to a decrease in the area of the Control colonies over the summer period, between the beginning and the end of the ladder experiment (Scheffé Post Hoc test, p-value=0.014). The decrease in the area of the branches was about 25% which was similar to that observed in the field[4]. Accordingly, the area of the Control colonies at the end of the experiment did not differ from that of the branches collected in fall (Scheffé Post Hoc test, p-value=0.989).

The fact that the area of the colonies used in the experiments displayed a decrease in area during the summer period similar to that observed to occur in the field, indicates that energy reserves of the colonies in the aquaria performed rather similar to those in the field, suggesting that experimental conditions for Control colonies may be considered suitable for the red gorgonian *Paramuricea clavata*.

Supplementary Methods S2. Further information ofPERMANOVA analyses.

We chose the non-parametric method of PERMANOVA because of the non-normal distribution of the dependent variable[3]. This method uses Euclidean distance as the basis of the multivariate analysis (see [4]) and relies on comparing the observed value of a test statistic (pseudo *F*-ratio) against a recalculated test statistic generated from random re-ordering (permutation) of the data[3]. Furthermore PERMANOVA was shown to be largely unaffected by heterogeneity of dispersions in the case of balanced designs[5].

In order to simplify the analyses, we avoided to include the Aquarium nested factor in the model because previous one-way PERMANOVA tests did not show statistical differences between treatment Aquariums (p-value>0.05 for all pair-wise comparisons). Accordingly, we pooled data from three replicates together to perform the analyses.

Instead of performing a repeated measure analysis of a single response variable, each time point was considered as a separate dependent variable (multivariate approach) because it completely avoided having to consider any notions of sphericity and interdependence of replicates[3].

Supplementary Methods S3. DNA extraction and microsatellite genotyping.

Total genomic DNA extraction of the 240 colonies belonging to the 8 populations was conducted based on the salting out procedure adapted from Miller *et al.*[6]. The colonies were genotyped using six microsatellites (Parcla-09, Parcla-10, Parcla-12, Parcla-14, Parcla-17 and Par-d) following[7]. PCR products were analyzed on an ABI 3130 Genetic Analyzer with GeneScan 600 LIZ internal size standard (Applied Biosystems). Genemapper version 3.0 (Applied Biosystems) was used to analyze the electrophoregrams. The occurrence of large allelic dropouts or scoring errors due to stuttering was checked with MICROCHECKER[8]. All the individuals that failed to amplify at more than 2 loci were deleted from the dataset. GIMLET[9] was used to remove repeated multilocus genotypes. Following analyses were conducted on 214 individuals. The frequencies of null alleles (*r*)were estimated for each population using the expectation maximization algorithm[10]in FREENA[11]. GENETIX 4.05[11]was used to estimate the total number of alleles per locus, the observed heterozygosity (H_o) and gene diversity (H_e;[12]), *f* estimator of F_{IS}[13] per sample, and to test linkage equilibrium for each pair of loci overall samples and in each sample using 1000 permutations. Departure from panmixia was tested for each sample using the score tests for heterozygote deficiency implemented in GENEPOP 4.1.4[14]. Significance of the result was computed by Markov Chain algorithm[15,16] using default parameters.

Supplementary Methods S4. R scritp for P_{ST} computation.

```
rm=ls()
library (vegan) data=read.csv("permanova_unif.csv",sep=";",dec=",",header=T) #data
data$Pop_cod=as.factor(data$Pop_cod)
data$Necrosis63=as.numeric(data$Necrosis63)
summary(data) #pre-treatment
c1_sh=subset(data, Pop=="c1_sh", select = c(Necrosis44,Necrosis54,Necrosis63,Pop,Pop_cod))
c1_d=subset(data, Pop=="c1_d", select = c(Necrosis44,Necrosis54, Necrosis63,Pop,Pop_cod))
c2=subset(data, Pop=="c2_sh", select = c(Necrosis44,Necrosis54, Necrosis63,Pop,Pop_cod))
c3=subset(data, Pop=="c1_sh", select = c(Necrosis44,Necrosis54, Necrosis63,Pop,Pop_cod))
m=subset(data, Pop=="Medes", select = c(Necrosis44,Necrosis54, Necrosis63,Pop,Pop_cod))
s=subset(data, Pop=="Sca", select = c(Necrosis44,Necrosis54, Necrosis63,Pop,Pop_cod))

#pairs
c1sh_c1d=rbind(c_sh,c_d)
c1sh_c1d$Pop_cod=as.factor(ifelse(c1sh_c1d$Pop=="c1_sh",1,2))
c1sh_m=rbind(c_sh,m)
c1sh_m$Pop_cod=as.factor(ifelse(c1sh_m$Pop=="c1_sh",1,2))
c1sh_s=rbind(c_sh,s)
c1sh_s$Pop_cod=as.factor(ifelse(c1sh_s$Pop=="c1_sh",1,2))
c1d_m=rbind(c_d,m)
c1d_m$Pop_cod=as.factor(ifelse(c1d_m$Pop=="c1_d",1,2))
c1d_s=rbind(c_d,s)
c1d_s$Pop_cod=as.factor(ifelse(c1d_s$Pop=="c1_d",1,2))
c2_c1sh=rbind(c2,c_sh)
c2_c1sh$Pop_cod=as.factor(ifelse(c2_c1sh$Pop=="c2_sh",1,2))
c2_c1d=rbind(c2,c_d)
c2_c1d$Pop_cod=as.factor(ifelse(c2_c1d$Pop=="c2_sh",1,2))
c2_m=rbind(c2,m)
c2_m$Pop_cod=as.factor(ifelse(c2_m$Pop=="c2_sh",1,2))
c2_s=rbind(c2,s)
c2_s$Pop_cod=as.factor(ifelse(c2_s$Pop=="c2_sh",1,2))
c3_c2=rbind(c3,c2)
c3_c2$Pop_cod=as.factor(ifelse(c3_c2$Pop=="c3_sh",1,2))
c3_c1sh=rbind(c3,c_sh)
c3_c1sh$Pop_cod=as.factor(ifelse(c3_c1sh$Pop=="c3_sh",1,2))
c3_c1d=rbind(c3,c_d)
c3_c1d$Pop_cod=as.factor(ifelse(c3_c1d$Pop=="c3_sh",1,2))
c3_m=rbind(c3,m)
c3_m$Pop_cod=as.factor(ifelse(c3_m$Pop=="c3_sh",1,2))
c3_s=rbind(c3,s)
c3_s$Pop_cod=as.factor(ifelse(c3_s$Pop=="c3_sh",1,2))
m_s=rbind(m,s)
m_s$Pop_cod=as.factor(ifelse(m_s$Pop=="Medes",1,2))

#first stage:
##for each pair of populations we do loops to generate 1000 samples with reposition of the
#multivariate dependent variable: (Necrosis44,Necrosis54,Necrosis63) #resampling must be: #1)by row
(considering the three variables together)
```

#2)inside each population

```
###########c3 - c2
a=c3[,1:3] RAND1a = sapply(1:1000,function(i)
{smp=a[sample(nrow(a),replace=T), ] })
A=matrix(0,30,3000)
 J=1000
for(j in 1:J) {
t=t(RAND1a[,j])
 tt=as.matrix(unlist(t))
A[1:30,3*j-2]=tt[1:30]
A[1:30,3*j-1]=tt[31:60]
A[1:30,3*j]=tt[61:90]
                                        }
b=c2[,1:3]
RAND1b = sapply(1:1000,function(i)
 {smp=b[sample(nrow(b),replace=T), ] })
B=matrix(0,30,3000)
 J=1000
             for(j in 1:J)           {
t=t(RAND1b[,j])
tt=as.matrix(unlist(t))
B[1:30,3*j-2]=tt[1:30]
B[1:30,3*j-1]=tt[31:60]
B[1:30,3*j]=tt[61:90]
                                        }
data1=rbind(A,B)
colnames(data1)= paste("rand",1:3000,sep="_")
Pop=c3_ph[,5]
data1=cbind(data1,Pop)

###########c3 - C-sh
a=c3[,1:3]
RAND1a = sapply(1:1000,function(i)
{smp=a[sample(nrow(a),replace=T), ] })
A=matrix(0,30,3000)
 J=1000
for(j in 1:J) {
 t=t(RAND1a[,j])
 tt=as.matrix(unlist(t))
A[1:30,3*j-2]=tt[1:30]
A[1:30,3*j-1]=tt[31:60]
A[1:30,3*j]=tt[61:90]    }
 b=c_sh[,1:3] RAND1b = sapply(1:1000,function(i)  {smp=b[sample(nrow(b),replace=T), ] })
B=matrix(0,30,3000)
J=1000  for(j in 1:J) {
t=t(RAND1b[,j])
tt=as.matrix(unlist(t))
B[1:30,3*j-2]=tt[1:30]
B[1:30,3*j-1]=tt[31:60]
```

```
B[1:30,3*j]=tt[61:90]     }
data2=rbind(A,B)
colnames(data2)= paste("rand",1:3000,sep="_")
Pop=c3_c1sh[,5] data2=cbind(data2,Pop)

########c3 - C-d
a=c3[,1:3]
RAND1a = sapply(1:1000,function(i)
 {smp=a[sample(nrow(a),replace=T), ] })
A=matrix(0,30,3000)
J=1000
for(j in 1:J) {
t=t(RAND1a[,j])
tt=as.matrix(unlist(t))
A[1:30,3*j-2]=tt[1:30]
A[1:30,3*j-1]=tt[31:60]
A[1:30,3*j]=tt[61:90]
}
b=c_d[,1:3]
RAND1b = sapply(1:1000,function(i)  {smp=b[sample(nrow(b),replace=T), ] })
B=matrix(0,30,3000)
J=1000
for(j in 1:J) {
t=t(RAND1b[,j])
tt=as.matrix(unlist(t))
B[1:30,3*j-2]=tt[1:30]
B[1:30,3*j-1]=tt[31:60]
B[1:30,3*j]=tt[61:90]
 }
data3=rbind(A,B)
colnames(data3)= paste("rand",1:3000,sep="_")
Pop=c3_c1d[,5]
data3=cbind(data3,Pop)
########c3 - Medes
 a=c3[,1:3]
RAND1a = sapply(1:1000,function(i)
{smp=a[sample(nrow(a),replace=T), ] })
A=matrix(0,30,3000)
 J=1000
 for(j in 1:J) {
 t=t(RAND1a[,j])
 tt=as.matrix(unlist(t))
A[1:30,3*j-2]=tt[1:30]
A[1:30,3*j-1]=tt[31:60]
A[1:30,3*j]=tt[61:90]    }
b=m[,1:3]
RAND1b = sapply(1:1000,function(i)
{smp=b[sample(nrow(b),replace=T), ] })
B=matrix(0,60,3000)
J=1000
```

```
for(j in 1:J) {
t=t(RAND1b[,j])
tt=as.matrix(unlist(t))
B[1:60,3*j-2]=tt[1:60]
B[1:60,3*j-1]=tt[61:120]
B[1:60,3*j]=tt[121:180]
}
data4=rbind(A,B)
colnames(data4)= paste("rand",1:3000,sep="_")
Pop=c3_m[,5] data4=cbind(data4,Pop)

########c3 - Sca
 a=c3[,1:3]
RAND1a = sapply(1:1000,function(i)
{smp=a[sample(nrow(a),replace=T), ] })
A=matrix(0,30,3000)
 J=1000
 for(j in 1:J) {
 t=t(RAND1a[,j])
tt=as.matrix(unlist(t))
A[1:30,3*j-2]=tt[1:30]
A[1:30,3*j-1]=tt[31:60]
A[1:30,3*j]=tt[61:90]  }
b=s[,1:3]
RAND1b = sapply(1:1000,function(i)
{smp=b[sample(nrow(b),replace=T), ] })
B=matrix(0,60,3000)
J=1000  for(j in 1:J) {
t=t(RAND1b[,j])
tt=as.matrix(unlist(t))
B[1:60,3*j-2]=tt[1:60]
B[1:60,3*j-1]=tt[61:120]
B[1:60,3*j]=tt[121:180]
 }
data5=rbind(A,B)
colnames(data5)= paste("rand",1:3000,sep="_")
Pop=c3_s[,5] data5=cbind(data5,Pop)

#######c2 - C-sh
a=c2[,1:3]
RAND1a = sapply(1:1000,function(i)
{smp=a[sample(nrow(a),replace=T), ] })
A=matrix(0,30,3000)
 J=1000
for(j in 1:J) {
 t=t(RAND1a[,j])
tt=as.matrix(unlist(t))
A[1:30,3*j-2]=tt[1:30]
A[1:30,3*j-1]=tt[31:60]
A[1:30,3*j]=tt[61:90]
```

```
}
b=c_sh[,1:3]
RAND1b = sapply(1:1000,function(i)
{smp=b[sample(nrow(b),replace=T), ] })
B=matrix(0,30,3000)
 J=1000
for(j in 1:J) {
 t=t(RAND1b[,j])
tt=as.matrix(unlist(t))
B[1:30,3*j-2]=tt[1:30]
B[1:30,3*j-1]=tt[31:60]
B[1:30,3*j]=tt[61:90]
}
 data6=rbind(A,B)
colnames(data6)= paste("rand",1:3000,sep="_")
Pop=c2_c1sh[,5]
data6=cbind(data6,Pop)
#######c2 - C-d
a=c2[,1:3]
RAND1a = sapply(1:1000,function(i)
{smp=a[sample(nrow(a),replace=T), ] })
A=matrix(0,30,3000)
J=1000
for(j in 1:J) {
t=t(RAND1a[,j])
tt=as.matrix(unlist(t))
A[1:30,3*j-2]=tt[1:30]
A[1:30,3*j-1]=tt[31:60]
A[1:30,3*j]=tt[61:90]    }
b=c_d[,1:3]
RAND1b = sapply(1:1000,function(i)
{smp=b[sample(nrow(b),replace=T), ] })
B=matrix(0,30,3000)
 J=1000
for(j in 1:J) {
t=t(RAND1b[,j])
tt=as.matrix(unlist(t))
B[1:30,3*j-2]=tt[1:30]
B[1:30,3*j-1]=tt[31:60]
B[1:30,3*j]=tt[61:90]     }
data7=rbind(A,B)
colnames(data7)= paste("rand",1:3000,sep="_")
Pop=c2_c1d[,5]
data7=cbind(data7,Pop)

########c2 - Medes
a=c2[,1:3]
RAND1a = sapply(1:1000,function(i)
{smp=a[sample(nrow(a),replace=T), ] })
A=matrix(0,30,3000)
```

```
J=1000
for(j in 1:J) {
t=t(RAND1a[,j])
tt=as.matrix(unlist(t))
A[1:30,3*j-2]=tt[1:30]
A[1:30,3*j-1]=tt[31:60]
A[1:30,3*j]=tt[61:90]    }
b=m[,1:3]
RAND1b = sapply(1:1000,function(i)
{smp=b[sample(nrow(b),replace=T), ] })
B=matrix(0,60,3000)
J=1000
for(j in 1:J) {
t=t(RAND1b[,j])
tt=as.matrix(unlist(t))
B[1:60,3*j-2]=tt[1:60]
B[1:60,3*j-1]=tt[61:120]
B[1:60,3*j]=tt[121:180]
}
 data8=rbind(A,B)
colnames(data8)= paste("rand",1:3000,sep="_")
Pop=c2_m[,5]
data8=cbind(data8,Pop)

########c2 - Sca
a=c2[,1:3]
RAND1a = sapply(1:1000,function(i)
{smp=a[sample(nrow(a),replace=T), ] })
A=matrix(0,30,3000)
J=1000  for(j in 1:J) {
t=t(RAND1a[,j])
tt=as.matrix(unlist(t))
A[1:30,3*j-2]=tt[1:30]
A[1:30,3*j-1]=tt[31:60]
A[1:30,3*j]=tt[61:90]
}
b=s[,1:3]
RAND1b = sapply(1:1000,function(i)
{smp=b[sample(nrow(b),replace=T), ] })
B=matrix(0,60,3000)
 J=1000
for(j in 1:J) {
t=t(RAND1b[,j])
tt=as.matrix(unlist(t))
B[1:60,3*j-2]=tt[1:60]
B[1:60,3*j-1]=tt[61:120]
B[1:60,3*j]=tt[121:180]
}
data9=rbind(A,B)
colnames(data9)= paste("rand",1:3000,sep="_")
```

```
Pop=c2_s[,5]
data9=cbind(data9,Pop)

#######C-sh - C-d
a=c_sh[,1:3]
RAND1a = sapply(1:1000,function(i)
{smp=a[sample(nrow(a),replace=T), ] })
A=matrix(0,30,3000)
 J=1000
for(j in 1:J) {
t=t(RAND1a[,j])
tt=as.matrix(unlist(t))
A[1:30,3*j-2]=tt[1:30]
A[1:30,3*j-1]=tt[31:60]
A[1:30,3*j]=tt[61:90]    }
b=c_d[,1:3]
RAND1b = sapply(1:1000,function(i)
{smp=b[sample(nrow(b),replace=T), ] })
B=matrix(0,30,3000)
J=1000
for(j in 1:J) {
t=t(RAND1b[,j])
tt=as.matrix(unlist(t))
B[1:30,3*j-2]=tt[1:30]
B[1:30,3*j-1]=tt[31:60]
B[1:30,3*j]=tt[61:90]
}
 data10=rbind(A,B)
colnames(data10)= paste("rand",1:3000,sep="_")
Pop=c1sh_c1d[,5]
data10=cbind(data10,Pop)

########C-sh - Medes
a=c_sh[,1:3]
RAND1a = sapply(1:1000,function(i)
{smp=a[sample(nrow(a),replace=T), ] })
A=matrix(0,30,3000)
 J=1000
for(j in 1:J) {
t=t(RAND1a[,j])
tt=as.matrix(unlist(t))
A[1:30,3*j-2]=tt[1:30]
A[1:30,3*j-1]=tt[31:60]
A[1:30,3*j]=tt[61:90]    }
b=m[,1:3]
RAND1b = sapply(1:1000,function(i)
{smp=b[sample(nrow(b),replace=T), ] })
B=matrix(0,60,3000)
J=1000
for(j in 1:J) {
```

```
t=t(RAND1b[,j])
tt=as.matrix(unlist(t))
B[1:60,3*j-2]=tt[1:60]
B[1:60,3*j-1]=tt[61:120]
B[1:60,3*j]=tt[121:180]
 }
data11=rbind(A,B)
colnames(data11)= paste("rand",1:3000,sep="_")
Pop=c1sh_m[,5]
data11=cbind(data11,Pop)

########C-sh - Sca
a=c_sh[,1:3]
RAND1a = sapply(1:1000,function(i)
{smp=a[sample(nrow(a),replace=T), ] })
A=matrix(0,30,3000)
 J=1000
for(j in 1:J) {
 t=t(RAND1a[,j])
tt=as.matrix(unlist(t))
A[1:30,3*j-2]=tt[1:30]
 A[1:30,3*j-1]=tt[31:60]
A[1:30,3*j]=tt[61:90]    }
b=s[,1:3]
RAND1b = sapply(1:1000,function(i)
{smp=b[sample(nrow(b),replace=T), ] })
B=matrix(0,60,3000)
 J=1000
for(j in 1:J) {
t=t(RAND1b[,j])
tt=as.matrix(unlist(t))
B[1:60,3*j-2]=tt[1:60]
B[1:60,3*j-1]=tt[61:120]
B[1:60,3*j]=tt[121:180]
}
 data12=rbind(A,B)
colnames(data12)= paste("rand",1:3000,sep="_")
Pop=c1sh_s[,5]
data12=cbind(data12,Pop)
########C-d - Medes
a=c_d[,1:3]
RAND1a = sapply(1:1000,function(i)
{smp=a[sample(nrow(a),replace=T), ] })
A=matrix(0,30,3000)
J=1000
for(j in 1:J) {
t=t(RAND1a[,j])
tt=as.matrix(unlist(t))
A[1:30,3*j-2]=tt[1:30]
A[1:30,3*j-1]=tt[31:60]
```

```
A[1:30,3*j]=tt[61:90]    }
b=m[,1:3]
RAND1b = sapply(1:1000,function(i)
{smp=b[sample(nrow(b),replace=T), ] })
B=matrix(0,60,3000)
 J=1000
for(j in 1:J) {
t=t(RAND1b[,j])
tt=as.matrix(unlist(t))
B[1:60,3*j-2]=tt[1:60]
B[1:60,3*j-1]=tt[61:120]
B[1:60,3*j]=tt[121:180]
 }
data13=rbind(A,B)
colnames(data13)= paste("rand",1:3000,sep="_")
Pop=c1d_m[,5]
data13=cbind(data13,Pop)

########c-d - Sca
a=c_d[,1:3]
RAND1a = sapply(1:1000,function(i)
{smp=a[sample(nrow(a),replace=T), ] })
A=matrix(0,30,3000)
 J=1000  for(j in 1:J) {
t=t(RAND1a[,j])
tt=as.matrix(unlist(t))
A[1:30,3*j-2]=tt[1:30]
A[1:30,3*j-1]=tt[31:60]
A[1:30,3*j]=tt[61:90]    }
b=s[,1:3]
RAND1b = sapply(1:1000,function(i)
{smp=b[sample(nrow(b),replace=T), ] })
B=matrix(0,60,3000)
 J=1000  for(j in 1:J) {
t=t(RAND1b[,j])
tt=as.matrix(unlist(t))
B[1:60,3*j-2]=tt[1:60]
B[1:60,3*j-1]=tt[61:120]
B[1:60,3*j]=tt[121:180]
}
data14=rbind(A,B)
colnames(data14)= paste("rand",1:3000,sep="_")
Pop=c1d_s[,5]
data14=cbind(data14,Pop)

########M - Sca
a=m[,1:3]
RAND1a = sapply(1:1000,function(i)
{smp=a[sample(nrow(a),replace=T), ] })
 A=matrix(0,60,3000)
```

```
J=1000
for(j in 1:J) {
t=t(RAND1a[,j])
tt=as.matrix(unlist(t))
A[1:60,3*j-2]=tt[1:60]
A[1:60,3*j-1]=tt[61:120]
A[1:60,3*j]=tt[121:180]
 }
b=s[,1:3]
RAND1b = sapply(1:1000,function(i)
{smp=b[sample(nrow(b),replace=T), ] })
B=matrix(0,60,3000)
J=1000
for(j in 1:J) {
t=t(RAND1b[,j])
tt=as.matrix(unlist(t))
B[1:60,3*j-2]=tt[1:60]
B[1:60,3*j-1]=tt[61:120]
B[1:60,3*j]=tt[121:180]    }
data15=rbind(A,B)
 colnames(data15)= paste("rand",1:3000,sep="_")
Pop=m_s[,5]
data15=cbind(data15,Pop)
data15

####LOOP WITH LIST
x=list(data1,data2,data3,data4,data5,data6,data7,data8,data9,data10,data11,data12,data13, data
14,data15)
#c/h2=1
PSTdata=matrix(0,15,3)
 J=15
for (j in 9:J) {
 aux=x[[j]]
K=1000
for(k in 1:K){
POP=aux[,3001]
cbind(POP,aux)
Y=aux[,3*k-2:3*k]
modelo=adonis(Y~POP,method="euclidean",permutations=999)
MSb=modelo$aov.tab[1,3]
MSw=modelo$aov.tab[2,3]
var_w=MSw
nr=nrow(aux)
a=1/((length(unique(POP)))-1)
No=a*(nr((((nrow(subset(aux,aux[,3001]==1)))^2)+((nrow(subset(aux,aux[,3001]==2)))^2))/nr))
var_b=max((MSb-MSw)/No,0)   PST[k]=(1*var_b) / ((1 * var_b) + (2 * var_w))
}
PSTdata[j,1]=median(PST)
PSTdata[j,2]=sort(PST)[25]
PSTdata[j,3]=sort(PST)[975]
```

```
 }  #MEDIAN AND 95% CI
PSTdata

####global PST################
a=c3[,1:3]
RANDc3 = sapply(1:1000,function(i)
{smp=a[sample(nrow(a),replace=T), ] })
A=matrix(0,30,3000)
J=1000
for(j in 1:J) {
 t=t(RANDc3[,j])
tt=as.matrix(unlist(t))
A[1:30,3*j-2]=tt[1:30]
A[1:30,3*j-1]=tt[31:60]
A[1:30,3*j]=tt[61:90]     }
b=c2[,1:3]
RANDc2 = sapply(1:1000,function(i)
{smp=b[sample(nrow(b),replace=T), ] })
B=matrix(0,30,3000)
J=1000  for(j in 1:J) {
 t=t(RANDc2[,j])
tt=as.matrix(unlist(t))
B[1:30,3*j-2]=tt[1:30]
B[1:30,3*j-1]=tt[31:60]
B[1:30,3*j]=tt[61:90]     }
c=c_sh[,1:3]
RANDc1sh = sapply(1:1000,function(i)
{smp=c[sample(nrow(c),replace=T), ] })
C=matrix(0,30,3000)
 J=1000
for(j in 1:J) {
t=t(RANDc1sh[,j])
tt=as.matrix(unlist(t))
C[1:30,3*j-2]=tt[1:30]
C[1:30,3*j-1]=tt[31:60]
C[1:30,3*j]=tt[61:90]    }
d=c_d[,1:3]
RANDc1d = sapply(1:1000,function(i)
{smp=d[sample(nrow(d),replace=T), ] })
D=matrix(0,30,3000)
 J=1000
for(j in 1:J) {
t=t(RANDc1d[,j])
tt=as.matrix(unlist(t))
D[1:30,3*j-2]=tt[1:30]
D[1:30,3*j-1]=tt[31:60]
D[1:30,3*j]=tt[61:90]
}
e=m[,1:3] RAND
m = sapply(1:1000,function(i)
```

```
{smp=e[sample(nrow(e),replace=T), ] })
E=matrix(0,60,3000)
J=1000
for(j in 1:J) {
t=t(RANDm[,j])
tt=as.matrix(unlist(t))
E[1:60,3*j-2]=tt[1:60]
E[1:60,3*j-1]=tt[61:120]
E[1:60,3*j]=tt[121:180]
}
f=s[,1:3]
RANDs= sapply(1:1000,function(i)
{smp=f[sample(nrow(f),replace=T), ] })
F=matrix(0,60,3000)
J=1000
for(j in 1:J) {
t=t(RANDs[,j])
 tt=as.matrix(unlist(t))
F[1:60,3*j-2]=tt[1:60]
F[1:60,3*j-1]=tt[61:120]
F[1:60,3*j]=tt[121:180]
}
DATA=rbind(A,B,C,D,E,F)
colnames(DATA)= paste("rand",1:3000,sep="_")
DATA=cbind(DATA,data[,5])
#c/h2=1
PST_DATA=matrix(0,1,3)
aux=DATA
K=1000
for(k in 1:K){
POP=aux[,3001]
#cbind(POP,aux)
Y=aux[,3*k-2:3*k]
modelo=adonis(Y~POP,method="euclidean",permutations=999)
MSb=modelo$aov.tab[1,3]
MSw=modelo$aov.tab[2,3]
var_w=MSw
nr=nrow(aux)
aa=1/((length(unique(POP)))-1)
No=aa*(nr-(((30^2)+(30^2)+(30^2)+(30^2)+(60^2)+(60^2))/nr))
 var_b=max(((MSb-MSw)/No),0)
PST[k]=(1*var_b) / ((1 * var_b) + (2 * var_w))
PST_DATA=median(PST)
PST_DATA5=sort(PST)[25]
PST_DATA95=sort(PST)[975]
 #MEDIAN AND 95% CI }
PST_DATA PST_DATA5 PST_DATA95
##RESULTS #> PST_DATA #[1] 0.2501283 #> PST_DATA5 #[1] 0.007503372 #> PST_DATA95 #[1]
0.4358266
####END############
```

Supplementary References S1. References of the supplementary information.

1. Coma, R. *et al.* Global warming-enhanced stratification and mass mortality events in the Mediterranean. *Proc. Natl. Acad. Sci.***106,** 6176–6181 (2009).
2. Linares, C., Cebrian, E., Kipson, S. & Garrabou, J. Does thermal history influence the tolerance of temperate gorgonians to future warming? *Mar. Environ. Res.***89,** 45–52 (2013).
3. Anderson, M. J. A new method for non-parametric multivariate analysis of variance. *Austral Ecol.***26,** 32–46 (2001).
4. Anderson, M. J., Gorley, R. N. & Clarke, K. R. *PERMANOVA + for PRIMER: guide to software and statistical methods. PRIMER-E.* (2008).
5. Anderson, M. J. & Walsh, D. C. I. PERMANOVA, ANOSIM, and the Mantel test in the face of heterogeneous dispersions: What null hypothesis are you testing? *Ecol. Monogr.***83,** 557–574 (2013).
6. Miller, S.A., Dykes, D.D. & Polesky, H.F. A simple salting out procedure for extracting DNA from human nucleated cells. *Nucleic Acids Res.***16,** 1215 (1998).
7. MOKHTAR-JAMAÏ, K. *et al.* From global to local genetic structuring in the red gorgonian *Paramuricea clavata*: the interplay between oceanographic conditions and limited larval dispersal. *Mol. Ecol.***20,** 3291–3305 (2011).
8. Van Oosterhout, C., Hutchinson, W.F., Wills, D.P.M. & Shipley, P. MICRO-CHECKER: software for identifying and correcting genotyping errors in microsatellite data. *Mol. Ecol. Notes***6,** 255–256 (2004).
9. Valière, N. GIMLET: a computer program for analyzing genetic individual identification data. *Mol. Ecol. Notes***2,** 377–379 (2002).

10. Dempster, A., Laird, N. & Rubin, D. Maximum likelihood from incomplete data via the EM algorithm. *J. R. Stat. Soc. Ser. B***39,** 1–38 (1977).

11. Belkhir, K., Borsa, P., Chikhi, L., Raufaste, N. & Bonhomme, F. *GENETIX 4.05, logiciel sous Windows TM pour la génétique des populations*. (Laboratoire Génome, Populations, Interactions, CNRS UMR 5000, 2004).

12. Nei, M. Analysis of gene diversity in subdivided populations. *Proc. Natl. Acad. Sci. U. S. Am.***70,** 3321–3 (1973).

13. Weir, B. S. & Cockerham, C. C. Estimating F-statistics for the analysis of population structure. *Evolution***38,** 1358–1370 (1984).

14. Rousset, F. genepop'007: a complete re-implementation of the genepop software for Windows and Linux. *Mol. Ecol. Resour.***8,** 103–106 (2008).

15. Guo, S. & Thompson, E. Performing the exact test of Hardy-Weinberg proportion for multiple alleles. *Biometrics***48,** 361–372 (1992).

16. Raymond, M. & Rousset, F. GENEPOP (ver. 1.2): a population genetics software for exact test and ecumenicism. *J. Hered.***86,** 248–249 (1995).
